# 3D Volumetric Modeling and Microvascular Reconstruction of Irradiated Lumbosacral Defects after Oncologic Resection

**DOI:** 10.3389/fsurg.2016.00066

**Published:** 2016-12-13

**Authors:** Emilio Garcia-Tutor, Marco Romeo, Michael P. Chae, David J. Hunter-Smith, Warren Matthew Rozen

**Affiliations:** ^1^Department of Plastic and Reconstructive Surgery, Hospital de Guadalajara, Guadalajara, Spain; ^2^MD Anderson Cancer Center, Madrid, Spain; ^3^Department of Surgery, School of Clinical Science at Monash Health, Faculty of Medicine, Monash University, Monash Medical Centre, Clayton, VIC, Australia; ^4^Monash University Plastic and Reconstructive Surgery Unit (Peninsula Clinical School), Peninsula Health, Frankston, VIC, Australia

**Keywords:** volumetric analysis, preoperative imaging, planning, free flap, model

## Abstract

**Background:**

Locoregional flaps are sufficient in most sacral reconstructions. However, large sacral defects due to malignancy necessitate a different reconstructive approach, with local flaps compromised by radiation and regional flaps inadequate for broad surface areas or substantial volume obliteration. In this report, we present our experience using free muscle transfer for volumetric reconstruction, in such cases, and demonstrate three-dimensional (3D) haptic models of the sacral defect to aid preoperative planning.

**Methods:**

Five consecutive patients with irradiated sacral defects secondary to oncologic resections were included, surface area ranging from 143–600 cm^2^. Latissimus dorsi (LD)-based free flap sacral reconstruction was performed in each case, between 2005 and 2011. Where the superior gluteal artery was compromised, the subcostal artery (SA) was used as a recipient vessel. Microvascular technique, complications, and outcomes are reported. The use of volumetric analysis and 3D printing is also demonstrated, with imaging data converted to 3D images suitable for 3D printing with Osirix software (Pixmeo, Geneva, Switzerland). An office-based, desktop 3D printer was used to print 3D models of sacral defects, used to demonstrate surface area and contour and produce a volumetric print of the dead space needed for flap obliteration.

**Results:**

The clinical series of LD free flap reconstructions is presented, with successful transfer in all cases, and adequate soft-tissue cover and volume obliteration achieved. The original use of the SA as a recipient vessel was successfully achieved. All wounds healed uneventfully. 3D printing is also demonstrated as a useful tool for 3D evaluation of volume and dead space.

**Conclusion:**

Free flaps offer unique benefits in sacral reconstruction where local tissue is compromised by irradiation and tumor recurrence, and dead space requires accurate volumetric reconstruction. We describe for the first time the use of the SA as a recipient in free flap sacral reconstruction. 3D printing of haptic bio-models is a rapidly evolving field with a substantial role in preoperative planning.

## Introduction

A vast majority of sacral wounds derived from pressure sores can be treated with either a local or regional flap, as conventionally taught (1). However, where wounds span a large area from oncological resection, have substantial volume deficits or involve areas compromised by radiotherapy, locoregional flaps are relatively contraindicated. Radiation induces hypocellularity and hypovascularity in the normal tissue surrounding the tumor compromising its vascularity and, hence, its viability as a flap option (2, 3). Furthermore, reconstruction of the irradiated lumbosacral defects is curtailed by the reduced number and caliber of the vessels at the recipient site. Moreover, the need to restore a composite defect and the proximity of the anal region potentially increases the infection risk and present significant challenges for reconstructive surgeons.

In recent times, reconstructive surgeons have benefited from the availability of three-dimensional (3D) haptic bio-models for preoperative planning and flap designing (4). These models are produced by rapid prototyping (RP) techniques that use scan data from the conventional imaging modalities, such as the computed tomography (CT) and the magnetic resonance imaging (MRI). RP has been utilized in industrial design for decades; however, it has been adopted for medical application only in the last two decades. RP has introduced a convenient method of fabricating physical 3D models that accurately represent the anatomical structures and provide a tactile, or haptic, feedback to the clinician facilitating a superior understanding of the spatial relationship between structures (5). In medicine, stereolithography and 3D printing are the most extensively studied RP techniques. In contrast to stereolithography, 3D printing is a newer technology that is more affordable, quicker, and more convenient (6). 3D-printed haptic models have demonstrated utility in numerous surgical disciplines, such as maxillofacial (6) and orthopedic surgery (7). In plastic and reconstructive surgery, they have been useful in flap designing for soft-tissue defects (8) and preoperative volumetric assessment of breast asymmetry (9).

The current paper reports our experience using a large voluminous free flap for a single-stage reconstruction of irradiated lumbosacral oncologic defects. The technique and surgical outcomes are assessed. The use of 3D haptic models of the sacral defect to aid preoperative planning and volumetric analysis is presented.

## Patients and Methods

A case series of five consecutive patients were included, each of which were planned for microvascular reconstruction of an irradiated sacral defect, between 2005 and 2011. The series comprised one female and four males, with a mean age of 41.5 (range: 32–69). Each patient presented with a malignancy in the sacral region that was reconstructed by the senior author (EGT) following oncologic resection and irradiation. The resection was either of a primary or a recurrent tumor and all patients received radiotherapy (Table 1). For each case, consideration of a muscle only, myocutaneous or fasciocutaneous flap was considered, with selection based on donor-site availability, volume filling and contour requirements. The specific flap selected in each case is described below.

**Table 1:** **Patient demographics and operative details**.

Case	Age	Sex	Pathology	Primary/recurrence	Recipient vessels	Immediate/delayed	Flap surface dimensions and area	Colostomy required	Complications	Follow-up (years)	Survival outcomes
1	69	Male	Squamous cell carcinoma	Recurrence	Superior gluteal artery (SGA)	Immediate	25 cm × 10 cm = 250 cm^2^	Yes	Nil	3	Long-term survival
2	38	Male	Squamous cell carcinoma	Primary	Subcostal artery (SA)	Immediate	18 cm × 20 cm = 360 cm^2^	Yes	Nil	3	Long-term survival
3	35	Female	Radiation-induced sarcoma	Recurrence	SGA	Immediate	11 cm × 13 cm = 143 cm^2^	No	Nil	9	Mortality
4	32	Male	Ewing’s sarcoma	Recurrence	SGA	Immediate	20 cm × 10 cm = 200 cm^2^	No	Nil	7	Long-term survival
5	54	Male	Squamous cell carcinoma	Primary	SA	Delayed	20 cm × 30 cm = 600 cm^2^	Yes	Wound dehiscence requiring local flap	5	Mortality

### Surgical Technique

In delayed reconstructions, the volume and soft-tissue deficit was mapped preoperatively, while in immediate cases, this was able to be estimated based on resection planning. The 2D images from CT and MRI were used for initial evaluation of the planned reconstruction. Preoperative planning also involved the oncologic surgeon in surgical and reconstructive planning, in planning for estimated resection volumes. Flap choice was thus achieved, and where a skin paddle was sought, surface area able to be rapidly and accurately achieved.

The average wound surface was 359 cm^2^ (range: 200–600 cm^2^). In two cases, the skin surface defect was small enough for the skin island of the flap to adequately fill the surface defect. In two cases, the surface defects were larger and skin grafting was necessary. In one patient, the defect surface width was too broad and required closure with a tensor fascia lata flap. Regarding wound depth and volume, the LD flaps provided adequate tissue to fill and obliterate the cavity in all cases.

Consideration of a muscle only, myocutaneous or fasciocutaneous flap was considered, with selection based on donor-site availability, volume filling, and contour requirements as evaluated from imaging and bio-models. Ultimately, muscle only or myocutaneous latissimus dorsi (LD) flaps were selected in each case. In all cases, there was a volume requirement that exceeded the filling achievable with a fasciocutaneous flap, and the added benefits the longest pedicle possible was considered. Originally, deep inferior epigastric artery perforator flaps were also considered but were not used ultimately utilized as options.

Whenever they were available (three cases), superior gluteal artery (SGA) was the first-choice recipient vessel. In other cases where the gluteal arteries were damaged during the tumor resection or discarded due to their proximity to the tumor (two cases), subcostal artery (SA) of the eleventh rib was selected. All flap arteries and veins were anastomosed to recipient vessels in an end-to-end fashion. The location of a diverting stoma was planned based on imaging preoperatively and undertaken for wound care and patient positioning considerations.

### Creation of a 3D-Printed Haptic Bio-Model

**Figure 1:**
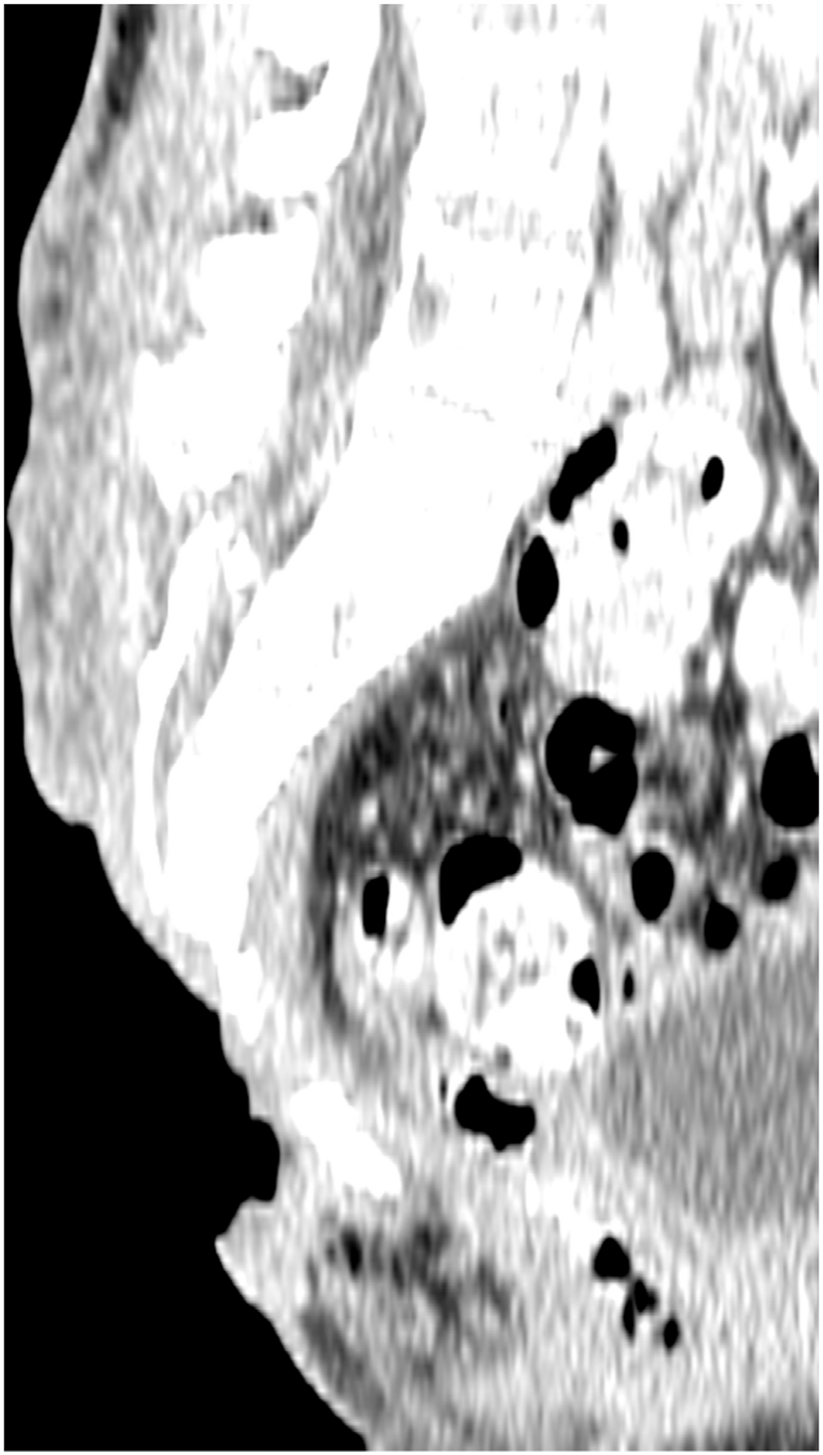
**Preoperative computed tomography scan, showing the three-dimensional nature of a sacral defect**.

**Figure 2:**
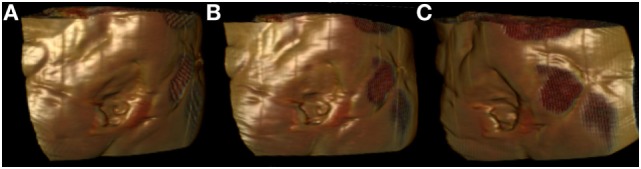
**Surface-rendered reconstruction images derived from a preoperative computed tomography scan, showing the three-dimensional nature of a sacral defect (A–C: three dimensional rotating views)**.

**Figure 3:**
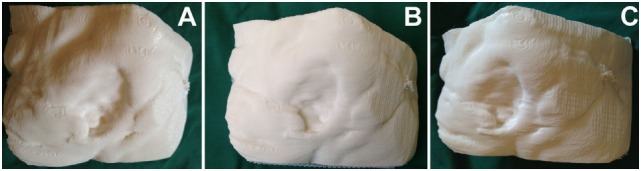
**Three-dimensional (3D) printed model of the sacral defect shown in Figure 2, produced using a 3D printer (Cube 2 printer, 3D Systems, Rock Hill, SC, USA) (A–C: three dimensional rotating views)**.

Imaging with either CT and/or MRI of the sacral region was used for volumetric analysis (see Figure 1). Haptic models of sacral defects were fabricated using a 3D printer similar to a technique described previously (9). The 2D scan data from CT or MRI was uploaded on to a computer using a free, third-party software called Osirix (Pixmeo, Geneva, Switzerland). The data were 3D-reconstructed using the “surface rendering” function (see Figure 2) and exported into a universal 3D file format called standard tessellation language (STL). The STL file was configured suitable for 3D printing using computer software, Cubify (3D Systems, Rock Hill, SC, USA), which accompanies the 3D printer (Cube 2 printer, 3D Systems). In Cubify (3D Systems), the model was reduced in size to fit within the maximum dimension of the printer (16 cm × 16 cm × 16 cm) and also orientated prone so that the defect was “pointing up” (see Figure 3). Due to the mechanism of 3D printing where the thermoplastic filament was deposited in a layer-by-layer fashion, where the defect was “pointing sideways,” the printer deposited a support structure to fill the “gap in height” (8). Although the support structures were easily removable, they left a rough surface that compromised the aesthetics of the model. The model was comparable to scan data for volumetric analysis, and the benefits of a 3D model with haptic feedback were assessed.

## Results

The LD free flap was successfully transferred in all cases (100% survival), with adequate soft-tissue cover and volume obliteration achieved. Each microvascular reconstruction with a free flap was performed in either the immediate post-resection setting or delayed if further irradiation or chemotherapy was planned. In the delayed cases, the open resected area was managed with local dressings and VAC therapy (KCI, San Antonio, TX, USA). In three cases, colostomy was necessary and presented an added advantage of preventing fecal contamination. Operative features of the patients are summarized in Table 1.

All wounds healed completely except one case, in which a minimal wound dehiscence occurred in the upper aspect of the sacrum defect, which required a lumbar artery perforator rotation flap to close. In three patients who had a colostomy, the wounds healed faster, especially in the caudal aspect of the flap near thee perianal region, and required less postoperative care. There were no wound infections and obliteration of the dead space and volumetric defect in all cases.

The anastomoses to the SGA and SA were performed without any major complications that necessitated an intraoperative or a postoperative revision of the flap. Of note, the anastomoses to the SGA differed to those to the SA due to their shorter and ramified pedicle, which arose vertically between the muscle fibers. At 3 years follow-up, three patients had survived. Two patients died within a year after the reconstruction secondary to tumor recurrence. All wounds had healed, with no donor-site complications and no need for secondary surgery.

Three-dimensional printing of sacral wound defects was achievable with either CT and/or MRI for all prints undertaken. The mean printing time for the scans was 12.5 h, and approximately 40% of the printer cartridge was required for each case (see Figure 3). The print was able to establish the cavity itself for haptic analysis, or able to be printed in a reverse manner, to demonstrate the volumetric defect itself.

### Case Examples

#### Case 1

**Figure 4:**
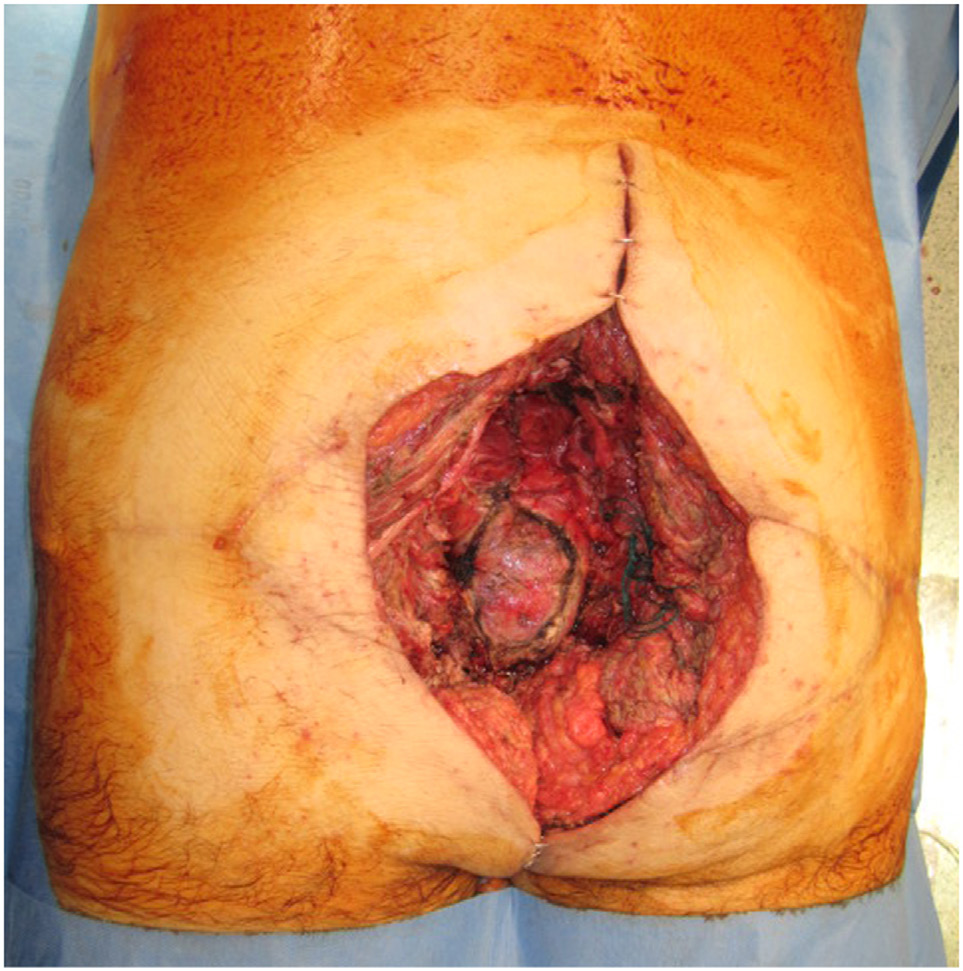
**A 25 cm × 10 cm surface area sacral defect requiring reconstruction**.

A 69-year-old man presented with a Marjolin’s ulcer secondary to a 20-year history of pilonidal cyst. Originally, the lesion was resected from another institution and reconstructed with a bilateral V-Y advancement flap. The patient represented a year later with extensive local recurrence. Preoperative imaging was performed with both CT and MRI. 3D volumetric analysis was undertaken with this imaging, and planned resection margins were recorded, able to preoperatively map the need for a 25 cm by 10 cm surface area defect and modeling highlighting a depth ranging from 10 to 13 cm. Resection planning thus highlighted the need for sacrectomy, colectomy, and a definitive colostomy with bladder exposure within ultimate the wound (see Figure 4).

**Figure 5:**
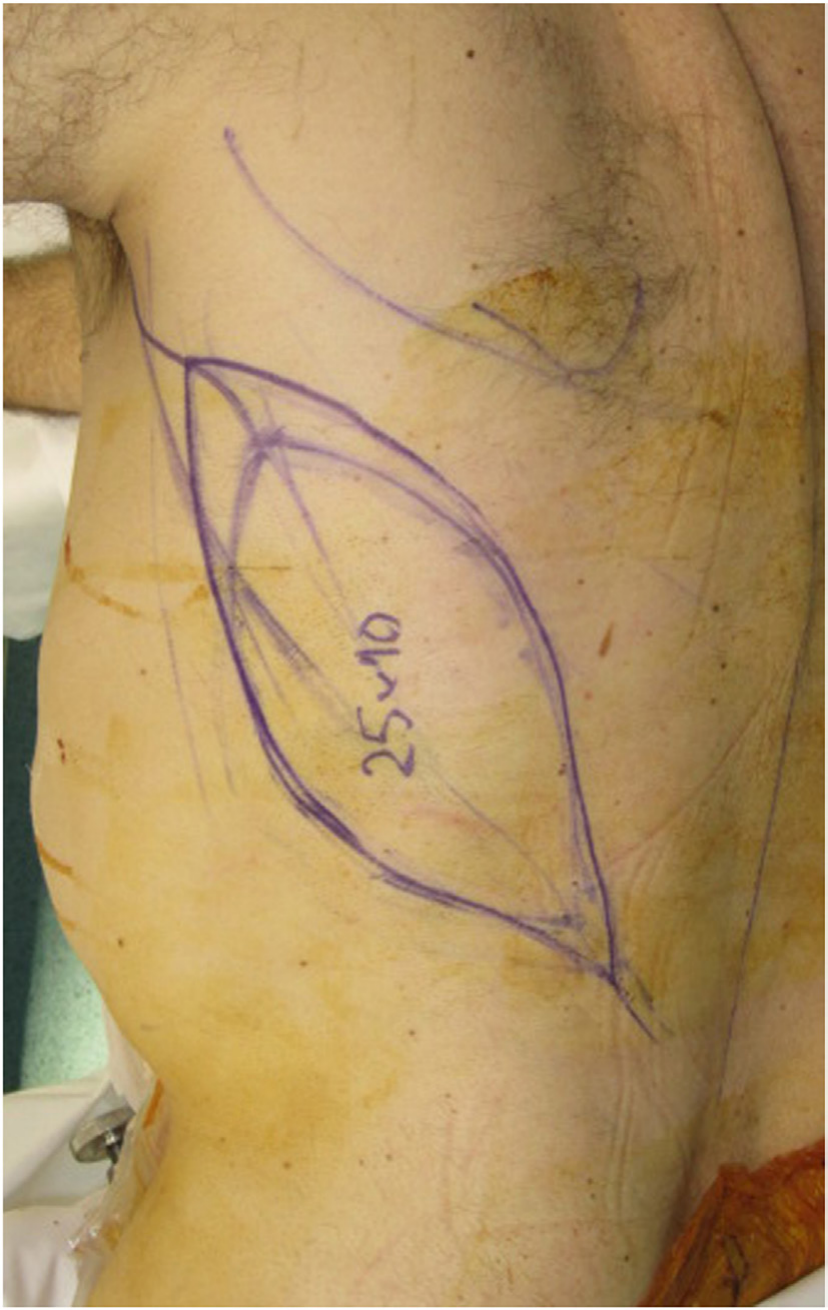
**A free latissimus dorsi myocutaneous flap was selected for reconstruction of the defect, with donor site marked**.

**Figure 6:**
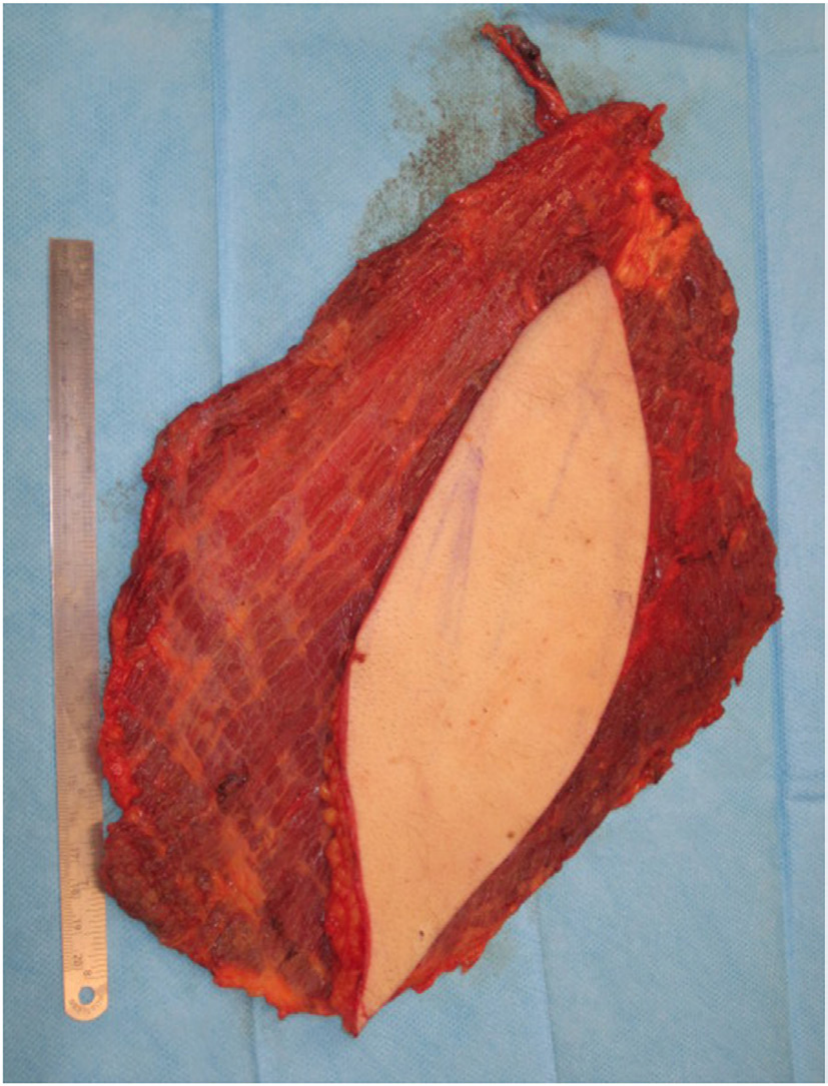
**Free latissimus dorsi myocutaneous flap harvested, with templated skin paddle and muscle for volumetric filling**.

**Figure 7:**
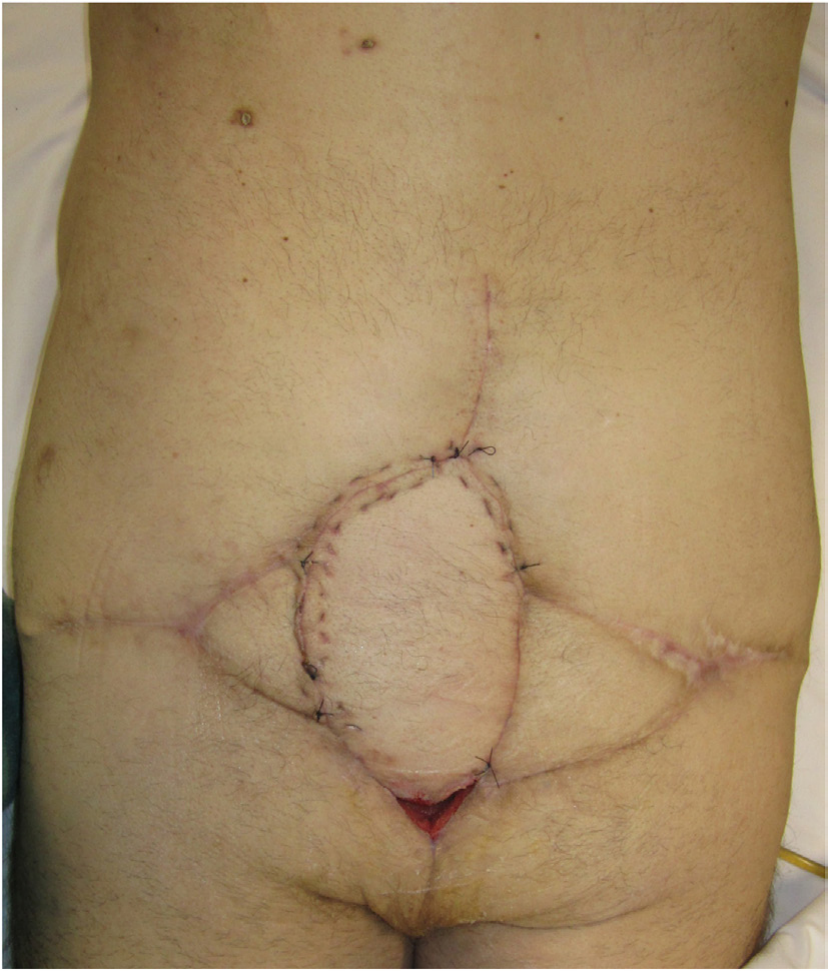
**Flap inset into sacral defect, with adjacent remnants of previous locoregional reconstructive flaps**.

A free LD flap with a 25 cm × 10 cm skin paddle was chosen for reconstruction (see Figure 5). The flap was harvested with the entirety of the muscle and skin paddle (see Figure 6), with arterial anastomosis performed to the SGA using an end-to-end technique and vein anastomosis performed using a Synovis microvascular coupler (Synovis Life Technologies, Birmingham, AL, USA). The flap was thus able to fill the 3D volumetric dead space and cover the defect (see Figure 7). There were no complications and good donor and recipient outcomes.

#### Case 2

A 38-year-old man presented with a Marjolin’s ulcer following a long-standing pilonidal cyst for 15 years. The lesion had been previously resected, including the cortical portion of the sacrum, and irradiated. The general surgeons planned for an immediate reconstruction, with resection and colostomy planned.

**Figure 8:**
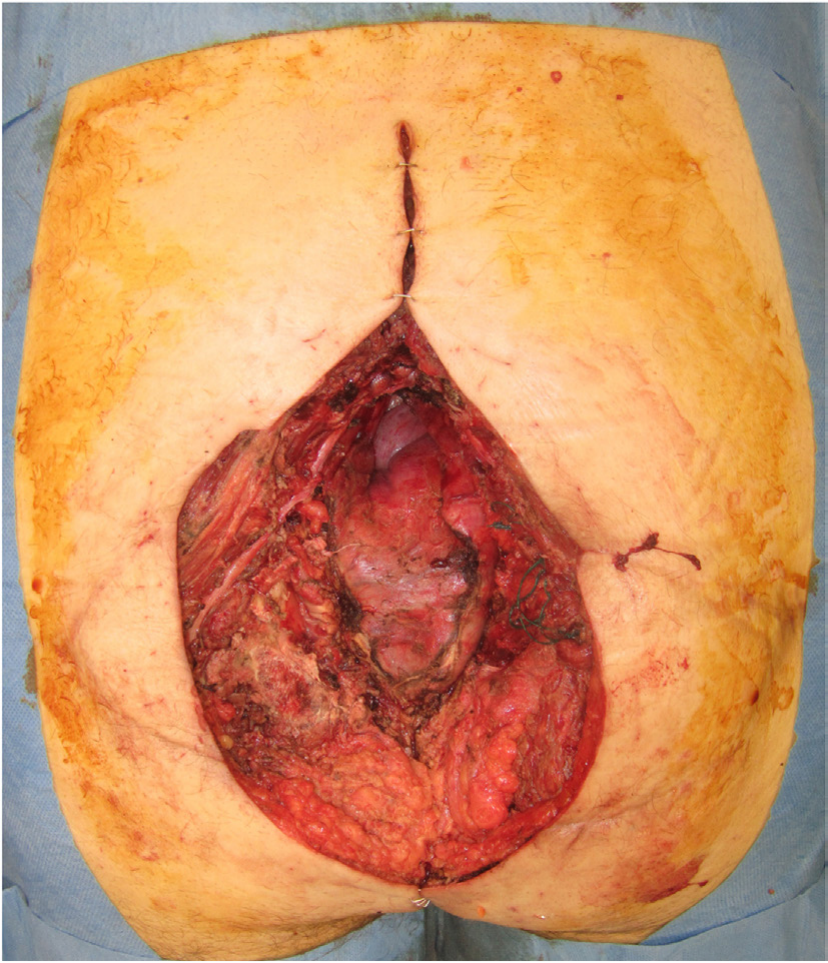
**An 18 cm × 20 cm surface area sacral defect requiring reconstruction**.

Preoperative imaging was performed with both CT and MRI. 3D volumetric analysis was undertaken with this imaging, and planned resection margins were recorded, with an 18 cm by 20 cm surface area defect planned and modeling highlighting a depth ranging from 5 to 11 cm. This closely mirrored the ultimate resection, where the defect comprised an 18 cm × 20 cm surface area defect, with substantial dead space volume (see Figure 8).

**Figure 9:**
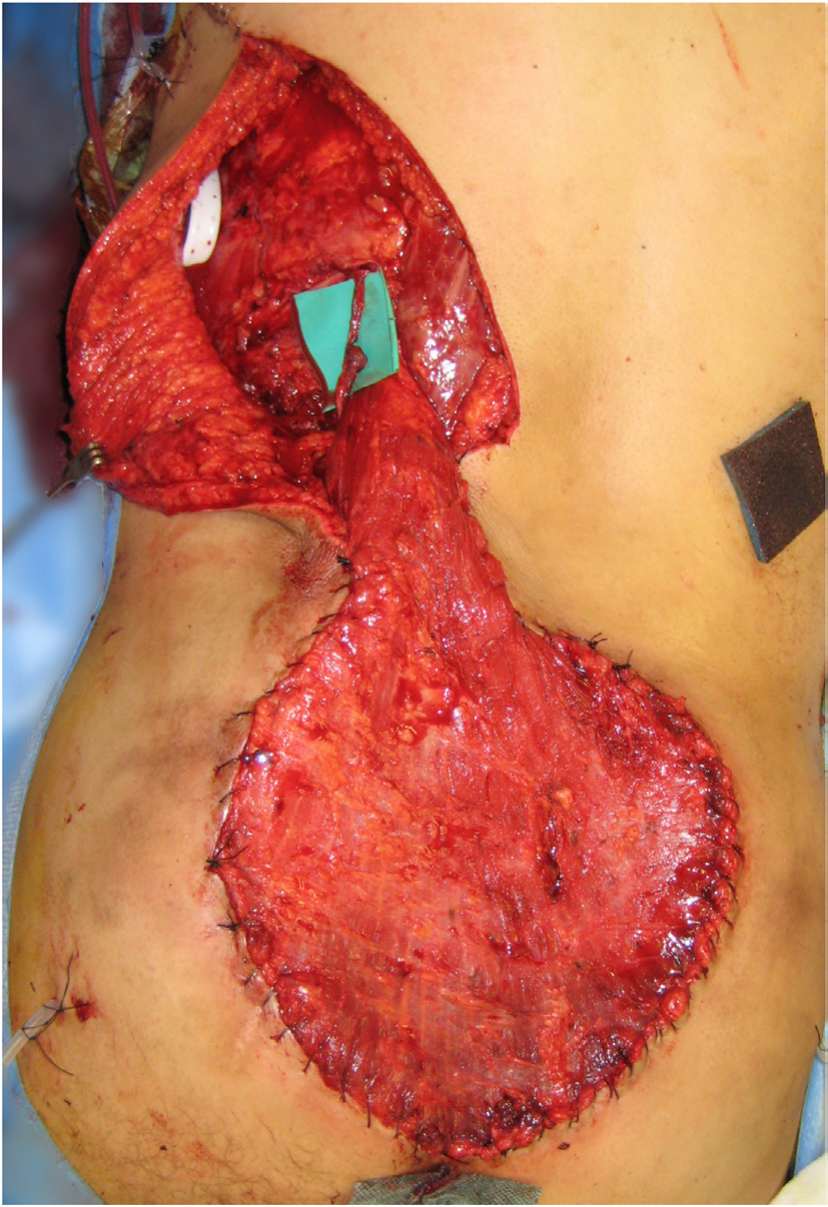
**Free latissimus dorsi muscle only flap inset into the defect**.

An LD muscle flap was chosen for reconstruction, and for the recipient vessel, the SA was chosen, since the SGA was too close to the resection area posing a risk of local recurrence and endangering the pedicle. The SA was exposed 15 cm cranial to the upper edge of the sacral defect, preventing the need for further operative morbidity. The vessels were anastomosed end-to-end, and despite some notable discrepancy in the caliber of donor and recipient veins, no complications eventuated. The flap suitably filled the defect (see Figure 9), was skin-grafted and healed uneventfully.

## Discussion

According to the traditional reconstructive ladder, microsurgical free flap repairs exist at the top. Recent advancements in preoperative imaging and operative techniques demand a re-ordering of the reconstructive ladder (10, 11). With 3D templating for soft-tissue reconstruction, a decision as to where to step on the ladder is made clearer. 3D analysis and volumetric planning have been used extensively and published broadly in a range of fields: breast reconstruction, cutaneous defects, and dead space filling (4, 6, 7). The ability to interact hands-on with the haptic bio-models that accurately represent the anatomical structures has revolutionized the way reconstructive surgeons would plan preoperatively and design free flaps (8, 9). In this field of sacral reconstruction, the specific dead space and volumetric needs of a reconstructive choice makes the technology of 3D analysis and 3D printing well-suited. The optimal choice of flap, approach to harvest and technique of inset to adequately replace volume and surface tissues can be achieved successfully.

One current limitation of this technology arises from having to outsource the fabrication of the bio-models to external companies that incur significant costs and a long production time. As the 3D printers become mainstream and more affordable, clinicians are able to quickly produce anatomical models at their own desktop (4, 8, 9, 12). The model used in this study was small-size replica of a sacral defect due to the maximal dimensions afforded by a desktop, office-based 3D printer (16 cm × 16 cm × 16 cm). Although the product was sufficiently detailed for the clinicians to appreciate the shape and depth of the defect, we expect to be able to print these lesions in life-size as the prices of larger 3D printers decrease. There are a range of office-based 3D printers available, the cheapest and the smallest of which make small models (16 cm × 16 cm × 16 cm), while there are other printers, such as MakerBot Z18 (MakerBot Industries, Brooklyn, NY, USA), that can print larger models (30 cm × 30 cm × 45 cm) exceeding the size of the defects described in this paper. We currently use both of these printers in our practice, and it is up to the individual surgeon to decide which is more preferred in terms of size, cost, and level of complexity. The smaller printer options are still useful in terms of comparative scale.

The current series comprises large defects, with substantial needs in volume and surface replacement. While locoregional flaps are the standard of care in most sacral wound defects, they are not plausible in many scenarios. Numerous local flap options have been reported, including gluteal artery perforator flaps (13–16), gluteus maximus sliding flaps (17), paraspinal flaps (18), V-Y advancement flaps (19, 20), and regional anterolateral thigh flaps (21). However, they are relatively contraindicated in oncological sacral defects. Local flaps are compromised by the collateral damage to the normal surrounding tissues from radiotherapy, and margins may not be completely clear of tumor spread. Regional flaps are disfiguring and may require more than one flap where a large defect needs to be covered, increasing donor-site morbidity. As a result, where a wide, heavily irradiated tissue area needs to be covered, locoregional flaps frequently necessitate multiple procedures. In contrast, there is currently insufficient evidence regarding the use of a free-tissue transfer for reconstruction of large complex defects.

From our case series, we demonstrate that microsurgical free flaps provide an alternative that is safe and facilitates a single-stage reconstruction that, as overall, results in a reduction in operative exposure. Conventional wisdom dictates that free flaps naturally require longer operative time due to their technical complexity. However, from our experience working with two surgical teams operating simultaneously on the donor site and the recipient site, there was a minimal difference between the operative length of a local flap and a free flap. Where wound dehiscence occurred, most commonly in the transitional area between the irradiated and the non-irradiated tissues, locoregional flaps have been useful as an adjunct.

Less discussion of free flaps for sacral reconstruction exist, with interpolation flaps utilizing omentum (22), the vertical rectus abdominis myocutaneous flap (23–25), the LD flaps, the chimeric LD flaps combined with the serratus anterior, and the free fibula flap all used in this setting (26, 27). Consistent with previous authors’ choice, the LD flap is reliable, easier, faster, and associated with less morbidities than many of the other options. Particularly for volumetric filling, LD flaps provide increased volume and depth by the ability to fold on themselves. Recipient vessels are of particular importance in sacral reconstruction, with superior and inferior gluteal, deep femoral and inferior epigastric arteries all considered (26, 27), often requiring intra-abdominal access or an arteriovenous loop (27). We describe an alternate option, the SA, which has consistent anatomy, few side branches and a long pedicle (up to 10 cm) (28). The use of a free flap based on the eleventh intercostal artery was first described by Badran et al. (29), and in 2006, Hamdi et al. classified the intercostal perforator flaps into dorsal, lateral or anterior depending on the portion of the SA being used (30).

## Conclusion

Free flaps may be a first-choice approach for sacral defects that are of substantial volume and have been irradiated. The relative lack of recipient vessels should not be a limitation, and we describe for the first time that the SA is a useful recipient in this setting. The volume of such defects can be assessed with traditional 2D imaging techniques, or with 3D-printed bio-models. Facilitated by modern preoperative planning technologies, 3D-printed haptic bio-models can map surface area and volume defects and may facilitate easier reconstruction planning.

## Ethics Statement

All 3D imaging and 3D printing was performed with approval of the Institutional Review Board, at Peninsula Health, Victoria, Australia. All patients signed informed consent statements. No other clinical trial registration was required, and no animal use or ethics was relevant. Biostatistics were performed as described in the methods. All data were anonymized, and no data sharing offered.

## Author Notes

Full authorship and ownership of the manuscript is with the authors above. The content of this article has not been submitted or published elsewhere. All the authors contributed significantly, and all the authors are in agreement with the content of the manuscript. The first and second authors’ contribution included manuscript preparation and literature review, the third and fourth authors were involved in all imaging aspects and manuscript preparation.

## Author Contributions

The authors of this manuscript have full ownership of the illustrations presented and permit their reproduction for the purposes of this publication. Both authors contributed equally and completely to the manuscript.

## Conflict of Interest Statement

The authors declare that there is no source of financial or other support, or any financial or professional relationships which may pose a competing interest.
